# Anti-dsDNA antibodies induce inflammation via endoplasmic reticulum stress in human mesangial cells

**DOI:** 10.1186/s12967-015-0536-7

**Published:** 2015-06-04

**Authors:** Hui Zhang, Chunmei Zhao, Shuang Wang, Yuefang Huang, Hongyue Wang, Jijun Zhao, Niansheng Yang

**Affiliations:** Department of Rheumatology, First Affiliated Hospital, Sun Yat-sen University, 58 Zhongshan Road II, Guangzhou, 510080 China; Department of Rheumatology, The First Affiliated Hospital of Xinjiang Medical University, 137 Liyushan South Road, Urumqi, 830054 China; Department of Pediatrics, First Affiliated Hospital, Sun Yat-sen University, 58 Zhongshan Road II, Guangzhou, 510080 China

**Keywords:** Anti-dsDNA antibodies, Endoplasmic reticulum stress, Inflammation, Human mesangial cell

## Abstract

**Background:**

Anti-dsDNA antibodies play an important role in the pathogenesis of lupus nephritis (LN). Endoplasmic reticulum (ER) stress is a physical reaction under stressful condition and can cause inflammation when stimulation is sustained. This study investigated the roles of ER stress in anti-dsDNA antibody-induced inflammation response in human mesangial cells (HMCs).

**Method:**

Anti-dsDNA antibodies isolated from LN patients were used to stimulate HMCs. The expression of GRP78, PERK, p-PERK, p-eIF2α, ATF4, p-IRE1α, ATF6 and CHOP in HMCs was measured by western blot. NF-κB activation was detected by examining nuclear translocation of NF-κB p65. The expression and production of IL-1β, TNF-α and MCP-1 were examined by qPCR and ELISA.

**Results:**

Flow cytometry and cellular ELISA showed that anti-dsDNA antibodies can bind to HMCs. The binding was not inhibited by blockage of Fc receptor. Anti-dsDNA antibody stimulation significantly enhanced the expression of GRP78, p-PERK, p-eIF2α and ATF4 in HMCs. However, no significant increase in the expression of p-IRE1α and ATF6 was found. In addition, anti-dsDNA antibodies also significantly increased the activation of NF-κB and upregulated the expression of IL-1β, TNF-α and MCP-1, which were suppressed by pretreatment of HMCs with chemical ER stress inhibitor 4-PBA. Transfection of specific ATF4 siRNA also significantly reduced the activation of NF-κB and expression of proinflammatory cytokines.

**Conclusion:**

Anti-dsDNA antibodies induce NF-κB activation and inflammation in HMCs via PERK-eIF2α-ATF4 ER stress pathway.

## Background

Systemic lupus erythematosus (SLE) is an autoimmune disease characterized by production of auto-antibodies [1]. Kidney is one of the most frequently involved organs in SLE. Deposition of auto-antibodies in the kidneys triggers inflammation resulting in lupus nephritis (LN), which may progress to end-stage renal failure [2].

Anti-double-stranded DNA (anti-dsDNA) antibodies are the hallmark auto-antibodies in SLE and correlated with disease activities of LN [3]. Previous study showed that monoclonal anti-dsDNA antibody stimulated the expression and release of inflammatory cytokines from normal human mononuclear cells [4]. Anti-dsDNA antibodies can also bind to human mesangial cell (HMCs) and cause inflammation [5]. However, the specific pathogenesis of anti-dsDNA antibodies in LN remains unclear.

Endoplasmic reticulum (ER) is recognized as a site for biosynthesis, folding, assembly, modification and degradation of proteins in the state of physiology [6]. ER stress is defined as accumulation of unfolded or misfolded proteins in the ER lumen. When the demand for ER function is over its capacity, ER stress arises [7]. In respond to ER stress, unfolded protein response (UPR) pathway was activated to prevent such accumulation in the ER lumen to cope with the stressful conditions [8, 9]. It is now recognized that three ER-localized transmembrane signal transducers including protein kinases IRE1 (inositol-requiring kinase 1), PERK (double-stranded RNA-activated protein kinase-like ER kinase), and the transcription factor ATF6 (activating transcription factor 6) can be activated to initiate adaptive responses in response to ER stress [10]. PERK is one of the three localized transmenbrane transducers. Released from Bip, PERK homodimerization and phosphorylation can phosphorylate eIF2α. The phosphorylation of eIF2α subsequently activates the transcription factor ATF4. At last, ATF4 induces the expression of UPR target genes, which are involved in oxidative stress response and inflammation [11].

Accumulating evidences indicate that ER stress has a close relation with inflammation and autoimmunity [12, 13]. NF-κB is a key transcription factor that has a central role in the initiation of inflammation [14]. NF-κB remains in cytoplasm in an inactive state and translocates into the nucleus once activated, inducing the transcription of numerous inflammation-associated genes [15]. It has been reported that NF-κB can be activated in response to ER stress [16], implying that ER stress might cause inflammation through activating NF-κB. In addition, the activation of NF-κB is involved in the pathogenesis of glomerulonephritis, including LN [17]. Extensive upregulation of NF-κB in renal tubular and interstitial cells was observed in LN [18].

It has been demonstrated that ER stress played important roles in glomerular and tubular damages in kidney diseases [19]. In experimental models of membranoproliferative glomerulonephritis and membranous nephropathy, ER stress has been described in glomerular cells [20]. However, the roles of ER stress in LN remained undefined. In this report, we demonstrated that anti-dsDNA antibodies from LN patients bound to HMCs, inducing ER stress and activated NF-κB through PERK-eIF2α-ATF4 ER stress pathway.

## Methods

### Patients and isolation of polyclonal anti-dsDNA antibodies or healthy serum IgG

Anti-dsDNA antibodies-positive sera were obtained from renal biopsy-proven active diffuse proliferative LN patients. Serum anti-dsDNA antibody level was measured and 10 serum samples with a concentration of anti-dsDNA antibody ≥4 IU/ml were used for this study. A concentration of 0–0.9 IU/ml was recognized as negative in this assay. The patient demographics were listed in Table 1. Twenty healthy sera were used as controls. Serum was obtained with informed consent with prior approval from the Institutional Ethical Committee of First Affiliated Hospital, Sun Yat-sen University. Polyclonal anti-dsDNA antibodies were isolated from sera of lupus patients and IgG was isolated from healthy subjects by affinity chromatography as previously described [21]. Immune complexes were removed from blood samples by polyethylene glycol precipitation before isolation. Serum samples were diluted with PBS to 1:1. Native DNA-cellulose column (GE Biotech, USA) was equilibrated with 25 mM Tris buffer (pH 8.0) at a flow rate of 0.5 ml/min and the diluted serum sample was added to the column. Non-DNA-binding fractions were flushed with the above buffer, and anti-DNA antibodies were eluted with a linear NaCl gradient. The columns were subsequently washed with 20 mM Tris–HCl (pH 7.4) containing 2 M NaCl, 1 mM ethylene diamine tetra acetic acid (EDTA), and 1 mM β-mercaptoethanol before further elution. IgG were then isolated with protein G Sepharose affinity chromatography kits (GE Biotech, USA), desalted and concentrated to 20-fold using ultrafree-4 centrifugal filter units (Millipore, USA). The purity of eluted IgG was confirmed by 10% sodium dodecyl sulfate–polyacrylamide gel electrophoresis. A NanoDrop spectrophotometer (Thermo Scientific, USA) was used to measure DNA level and no DNA contamination was detected in the isolated anti-dsDNA antibodies or control IgG.

**Table 1:** Patient demographics

Patients	Age	Sex	SLEDAI	Anti-dsDNA(IU/ml)
1	46	F	18	5.3
2	36	F	16	4.7
3	43	F	18	5.2
4	24	M	22	4.2
5	52	F	21	4.8
6	26	F	17	4.6
7	18	F	20	5.3
8	27	F	22	5.9
9	36	F	16	4.6
10	28	F	18	4.7

*F* female, *M* male, *SLEDAI* systemic lupus erythematosus disease activity index and *anti-dsDNA* anti-double strand DNA antibodies.

### Cell culture

HMCs (Science II, USA) were cultured in RMPI1640 culture medium containing 10% fetal bovine serum (FBS). Cells were maintained at 37°C in 5% CO_2_ in a humidified cell culture incubator. The media were changed every other day. HMCs were used for all experiments when cultured up to 90% confluence and incubated with serum-free medium for up to 12 h prior to use. Cells were plated in 60 mm culture dishes and stimulated with 100 nM of thapsigargin (TG, Sigma, USA), 10 μg/ml of anti-dsDNA antibodies, 10 μg/ml of normal human IgG, or medium alone for selected duration. In some experiments, 10 mM of 4-PBA (Sigma, USA) was included. The number of viable cells was assessed by trypan blue (trypan blue exclusion >90%).

### Detection the binding of anti-dsDNA antibodies to HMCs by flow cytometry

HMCs were grown up to 90% confluence and harvested. Cells (1 × 10^6^) were incubated with 20 μg/ml of anti-dsDNA antibodies from LN patients or IgG from healthy control at 4°C for 30 min, with or without blocking Fc receptors with anti-CD16/CD32/CD64 antibodies before incubation. After washed twice, cells were stained with FITC-conjugated anti-human IgG antibody (Abclonal, USA) at 4°C for 30 min. Cells were washed, resuspended in 500 μl staining buffer and analyzed by flow cytometry.

### Measurement of the binding of anti-dsDNA antibodies to HMCs by cellular ELISA

HMCs were seeded in 96-well culture plates until 90% confluence. Cells were fixed with 4% formaldehyde at room temperature for 10 min and blocked with 5% BSA at 37°C for 30 min. Then the cells were incubated with hydrogen peroxide solution at room temperature for 30 min. Cells were incubated with 20 μg/ml of anti-dsDNA antibodies from LN patients or IgG from healthy control at 4°C overnight, with or without blocking Fc receptors with anti-CD16/CD32/CD64 antibodies before incubation. Cells were washed and incubated with anti-human horseradish peroxidase conjugated-antibody for 30 min at room temperature. After washing, cells were incubated with o-phenylenediamine (OPD) and OD values were determined at 450 nm.

### Transfection

HMCs were plated in 60-mm dishes, cultured until 50% confluence and transfected with 40 nM ATF4 siRNA (Ruibo-bio, China) complexed with Lipofectamine 2000 (Invitrogen, USA) in 500 μl Opti-MEM I Reduced-Serum Medium (Invitrogen, USA) at 37°C in a CO_2_ incubator. In control experiments, cells were transfected with 40 nM of negative control siRNA complexed with Lipofectamine 2000. After 6 h of incubation, the RNAi-Lipofectamine complex was removed, and the cells were cultured overnight in 1640 supplemented with 10% FBS. Twenty-four hours after transfection, cells were grow-arrested for 12 h and maintained with serum-free medium for 24 h prior to use. Then HMCs were incubated with 10 μg/ml anti-dsDNA antibodies for 24 h.

### Western blot analysis

Proteins were extracted from HMCs and separated by 10% SDS–polyacrylamide gels. Nuclear and cytoplasmic proteins were extracted by using a nuclear protein extraction kit (Pierce, USA) according to the manufacture’s instructions. Then the proteins were electrotransferred onto polyvinylidinedifluoride membranes. After blocking with 5% bovine serum albumin in TBST, the membranes were incubated with anti-GRP78 (Novus, USA), anti-p-PERK (Cell Signaling Technology, USA), anti-PERK (Santa, Cruz, USA), anti-ATF4 (Santa Cruz, USA), anti-p-eIF2α (Cell Signaling Technology, USA), anti-eIF2α (Santa Cruz, USA), anti-IRE1α (Abcam, Hongkong), anti-ATF6 (Santa Cruz, USA), anti-CHOP (Santa Cruz, USA), anti-GAPDH (Kangcheng, China) primary antibodies at 4°C overnight. Nuclear protein was used to measure NF-κB p65 (Santa Cruz, USA) in the nucleus. Anti-fibrillarin (Santa Cruz, USA) was used as control. The membranes were then washed with TBST and incubated with horseradish peroxidase conjugated anti-rabbit IgG or anti-mouse IgG (Cell Signaling Technology, USA) at room temperature for 60 min, washed with TBST and the signal was detected by enhanced chemiluminescence (ECL). Band density was measured by densitometry with image J software.

### qPCR

Total RNA was extracted from cultured cell using Trizol Reagent (Invitrogen, USA). RNA was reverse-transcribed into cDNA using the revert transcriptase (Fermentas, USA). cDNA was amplified in a PCR reaction using recombinant Taq DNA polymerase (Fermentas, USA). The following primers for human MCP-1, TNF-α, IL-1β and GAPDH were used: MCP-1: 5′-GAT CTC AGT GCA GAG GCT CG-3′ (forward), 5′-TGC TTG TCC AGG TGG TCC AT-3′ (reverse), TNF-α: 5′-CCC AGG GAC CTC TCT CTA ATC A-3′ (forward), 5′-GCT ACA GGC TTG TEA CTC GG-3′ (reverse), IL-1β: 5′-CGT CAG TTG TTG TGG CCA T-3′ (forward), 5′-GCG TGC AGT TCA GTG ATC GTA-3′ (reverse), GAPDH:5′-GAA GGT GAA GGT CGG AGT C-3′ (forward), 5′- GAA GAT GGT GAT GGG ATT TC-3′ (reverse). SYBR green-based quantitative real-time PCR were performed in triplicate in a Bio-Rad IQ5. The cDNA were denatured at 95°C for 10 min and 35 cycles at 95°C for 15 s, 60°C for 1 min. Results of comparative real-time PCR were analyzed using IQ5 Software (Bio-Rad, USA). GAPDH was performed as an intracellular control to confirm the quantity of RNA.

### ELISA

HMCs were incubated with 10 μg/ml anti-dsDNA antibodies or control IgG for up to 48 h, after which the supernatant was collected and centrifuged at 2,000 rpm for 10 min to remove cell debris. The levels of IL-1β, MCP-1 and TNF-α in each sample were measured using commercial ELISA kits (Raybiotech, USA) according to the manufacturer’s instructions.

### Statistical analyses

The results are expressed as the mean ± SD. Statistical analysis was performed using SPSS 13.0. The differences were assessed by *t* test, or one way ANOVA with or without repeated measurements followed by Bonferroni’s multiple comparison post test as appropriate. Two-tailed *p* < 0.05 was considered statistically significant.

## Results

### Anti-dsDNA antibodies bound to HMCs

**Figure 1:**
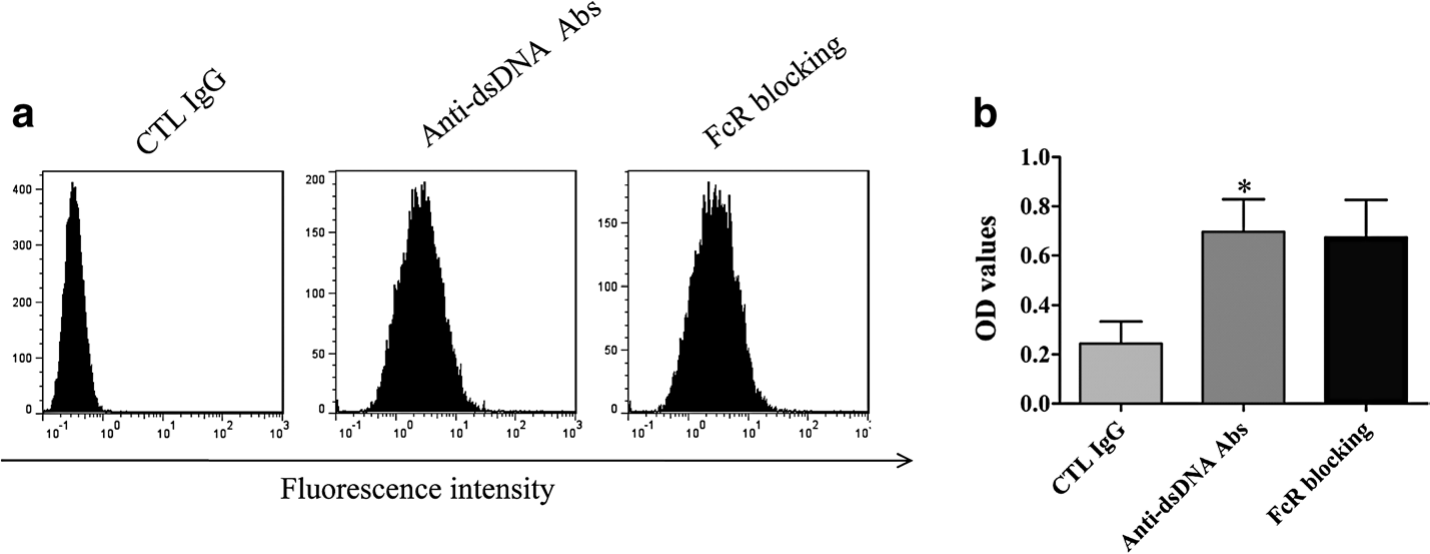
Anti-dsDNA antibodies bind to HMC. **a** HMCs were incubated with anti-dsDNA antibodies or control IgG, washed and stained with FITC conjugated anti-human IgG, with or without Fc receptor blocking. Flow cytometry analysis showed that the binding of anti-dsDNA antibodies to HMCs was not inhibited by Fc receptor blocking. **b** HMCs were incubated with anti-dsDNA antibodies or control IgG, washed and stained with HRP-conjugated anti-human IgG, with or without Fc receptor blocking. The binding of anti-dsDNA antibodies to HMCs was measured by cellular ELISA. No significant decrease in binding was observed after Fc receptor blocking. All experiments were repeated for three times. Data was expressed as mean ± SD, *P < 0.05 versus control IgG. *CTL* Control, *anti-dsDNA Abs* anti-dsDNA antibodies, and *FcR * Fc receptor.

Flow cytometry showed that anti-dsDNA antibodies bound to HMCs, while healthy control IgG from healthy individuals did not bind to HMCs (Figure 1a). To clarify the binding mechanism of anti-dsDNA antibodies to HMCs, Fc receptors on HMCs were blocked with anti-CD16/CD32/CD64 antibodies before incubated with anti-dsDNA antibodies. The binding of anti-dsDNA antibodies to HMCs was not inhibited by Fc receptor blocking (Figure 1a). The binding capacity of anti-dsDNA antibodies to HMCs was further confirmed by cellular ELISA (Figure 1b).

### Anti-dsDNA antibodies induced the activation of PERK ER stress pathway in HMCs

**Figure 2:**
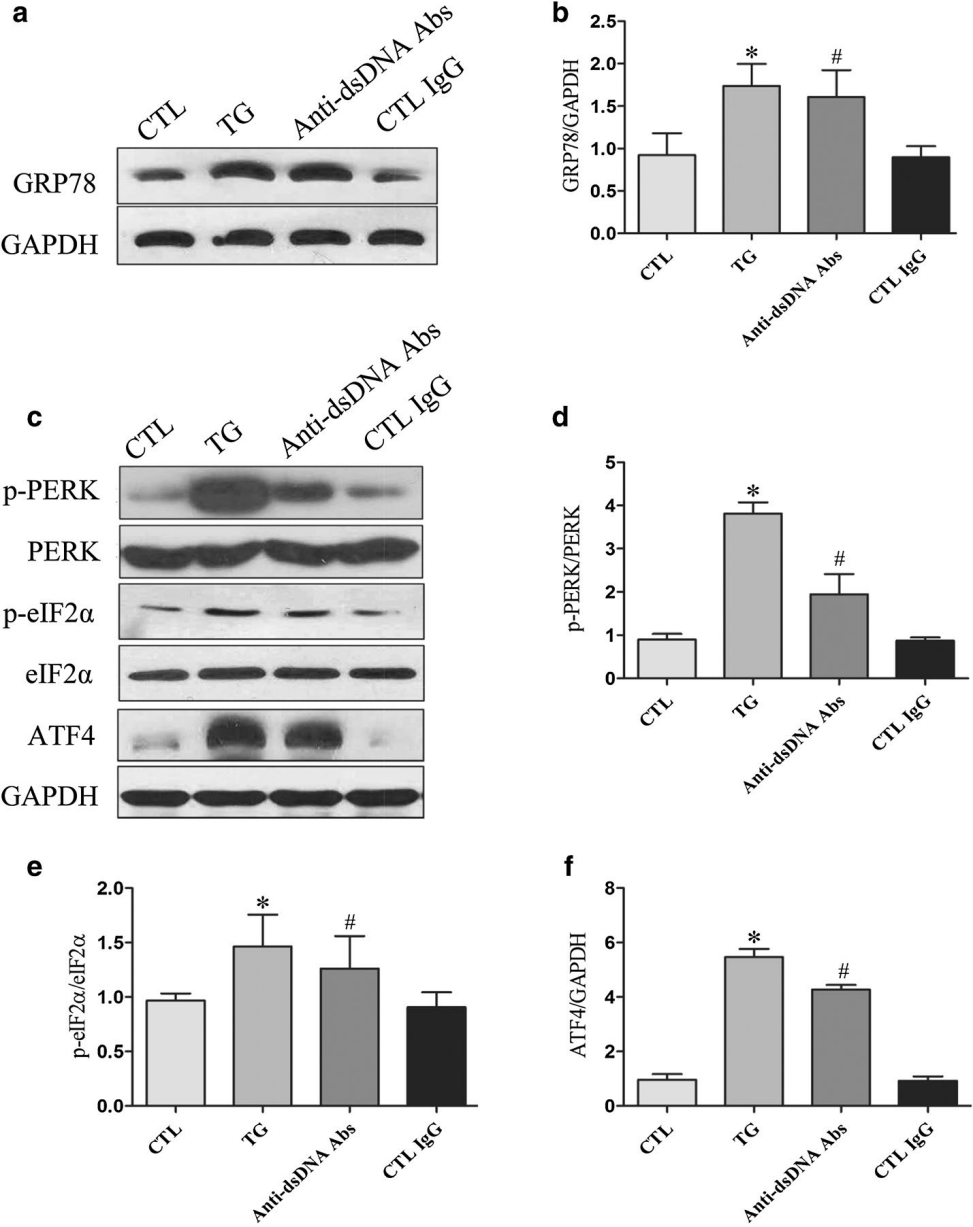
Anti-dsDNA antibodies induce ER stress in HMC. HMCs were seeded in 6-well culture plate and stimulated with anti-dsDNA antibodies for 24 h. **a** The expression of GRP78 was analyzed by western blot and **b** the relative expression of GRP78 was significantly increased when HMCs were stimulated with TG or anti-dsDNA antibodies. **c** HMCs were stimulated with anti-dsDNA antibodies for 24 h and the expression of p-PERK, p-eIF2α and ATF4 were analyzed by western blot. **d**–**f** Semi-quantificative analysis showed significant increase in the expression of p-PERK, p-eIF2α and ATF4. All experiments were repeated for three times. Data were expressed as mean ± SD, *P < 0.05, versus control media; #P < 0.05, versus control IgG. *CTL* Control, *anti-dsDNA Abs* anti-dsDNA antibodies and *TG* thapsigargin.

To study the effects of anti-dsDNA antibodies on HMCs, cells were cultured with anti-dsDNA antibodies (10 μg/ml), control IgG or TG (100 nM) for 24 h. Cells viability was assessed by trypan blue (trypan blue exclusion >90%). The expression of ER stress specific protein GRP78 was significantly up-regulated in HMCs stimulated with ER stress inducer thapsigargin (Figure 2a). Anti-dsDNA antibodies significantly enhanced the expression of GRP78, p-PERK, p-eIF2α, and ATF4 in HMCs compared to control IgG (Figure 2a, c). Semi-quantificative analysis showed significantly higher expression of GRP78, p-PERK, p-eIF2α, and ATF4 in HMCs stimulated with anti-dsDNA antibodies compared to cells incubated with control IgG (Figure 2b, d–f).

### Anti-dsDNA antibodies did not induce the activation of IRE1α and ATF6 ER stress pathways in HMCs

**Figure 3:**
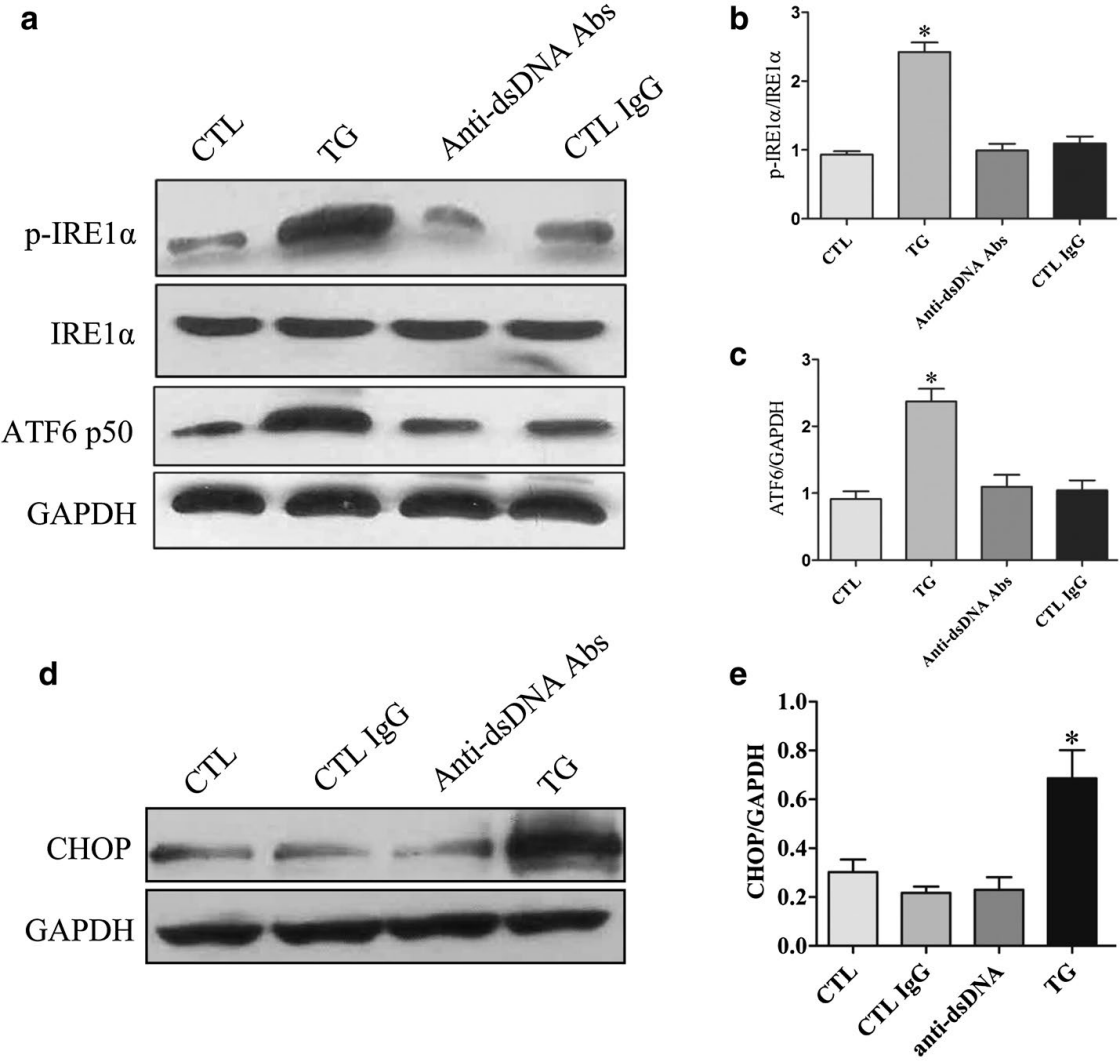
IRE1α and ATF6 pathways were not activated by anti-dsDNA antibodies in HMCs. HMCs were stimulated with anti-dsDNA antibodies for 24 h. **a** The expression of p-IRE1α and ATF6 were measured by western blot. **b**, **c** Semi-quantitative analysis showed no significant changes in the expression of p-IRE1α and ATF6. **d** The expression of CHOP was measured by western blot. **e** TG treatment significantly increased the expression of CHOP but no significant up-regulation of CHOP was found in MHCs stimulated with anti-dsDNA antibodies. All experiments were repeated for three times. Data were expressed as mean ± SD, *P < 0.05, versus control media. *CTL* Control, *anti-dsDNA Abs* anti-dsDNA antibodies and *TG* thapsigargin.

HMCs were incubated with anti-dsDNA antibodies, control IgG or TG for 24 h. Neither anti-dsDNA antibodies nor control IgG increased the expression of p-IRE1α and ATF6 in HMCs (Figure 3a). Semi-quantificative analysis showed no significant difference in the expression of p-IRE1α and ATF6 between HMCs stimulated with anti-dsDNA antibodies and cells incubated with control IgG (Figure 3b, c). Thapsigargin significantly increased the expression of CHOP in HMCs, but anti-dsDNA antibodies did not significantly increase the expression of CHOP in HMCs compared to control IgG (Figure 3d, e).

### Anti-dsDNA antibodies activated NF-κB and up-regulated inflammation in HMCs

**Figure 4:**
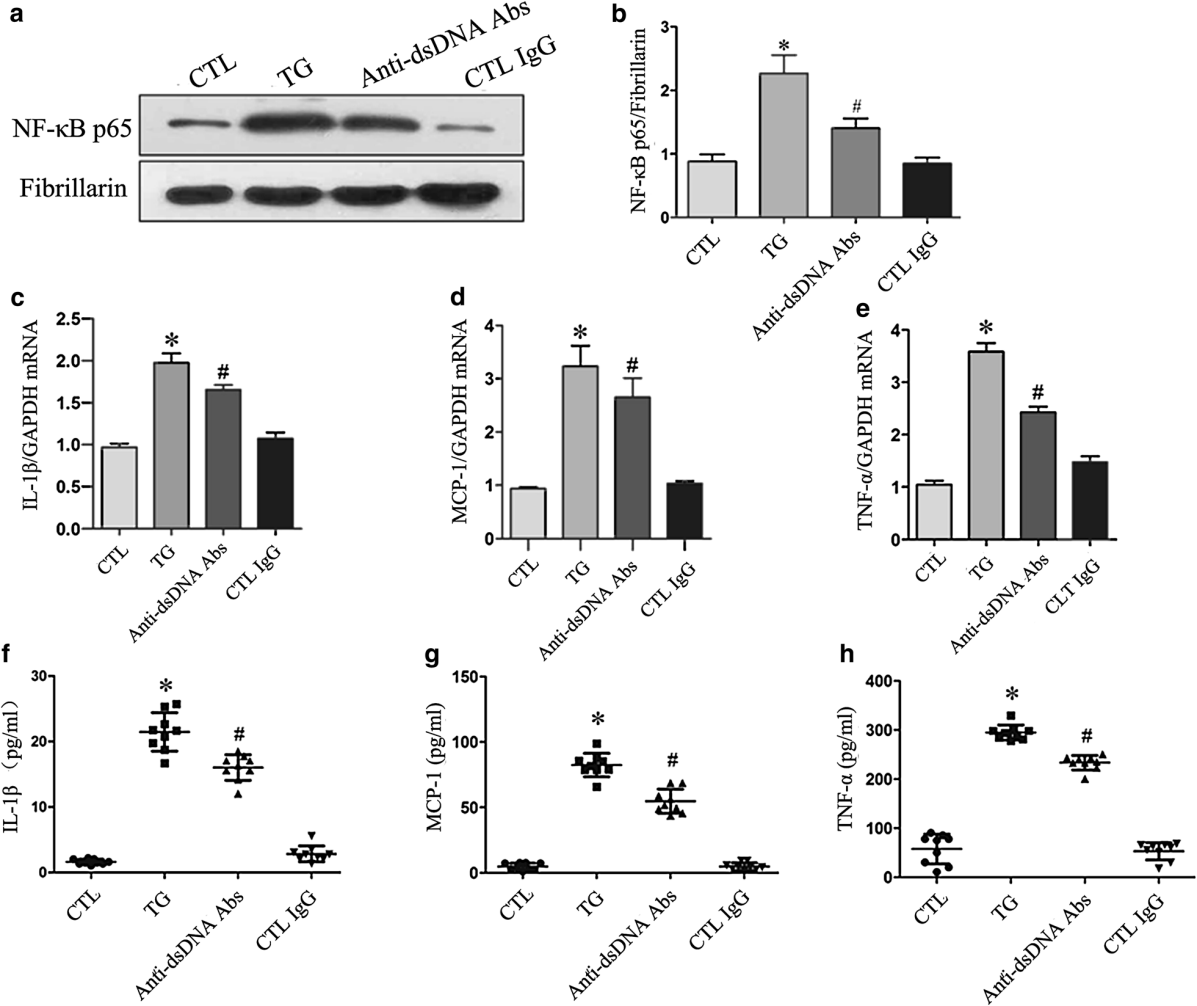
Anti-dsDNA antibodies activated NF-κB and caused inflammation in HMCs. HMCs were stimulated with anti-dsDNA antibodies. **a** NF-κB p65 was measured by western blot analysis of the nuclear protein extract. **b** Anti-dsDNA antibodies significantly increased nuclear translocation of NF-κB p65 compared to control IgG. **c**–**e** Anti-dsDNA antibodies significantly increased the expression of IL-1β, MCP-1 and TNF-α mRNA as measured by qPCR. **f**–**h** Anti-dsDNA antibodies also significantly increased the secretion of IL-1β, MCP-1 and TNF-α in supernatants as measured by ELISA. All experiments were repeated for three times. Data were expressed as mean ± SD, *P < 0.05, versus control media, #P < 0.05, versus control IgG. *CTL* Control, *anti-dsDNA* *Abs* anti-dsDNA antibodies and *TG* thapsigargin.

The expression NF-κB p65 was significantly increased in the nuclear extract of HMCs treated with thapsigargin, indicating that ER stress can induce the activation of NF-κB. Similarly, anti-dsDNA antibodies induced the nuclear translocation of NF-κB p65 in HMCs (Figure 4a). However, there was no significant increase in translocation of NF-κB p65 in HMCs stimulated with control IgG. The anti-dsDNA antibodies also significantly enhanced the gene expresses of IL-1β, TNF-α, MCP-1 (Figure 4c–e) and promoted the secretion of IL-1β, TNF-α and MCP-1 in supernatants (Figure 4f–h).

### Chemical chaperone inhibited anti-dsDNA antibody-induced ER stress in HMCs

**Figure 5:**
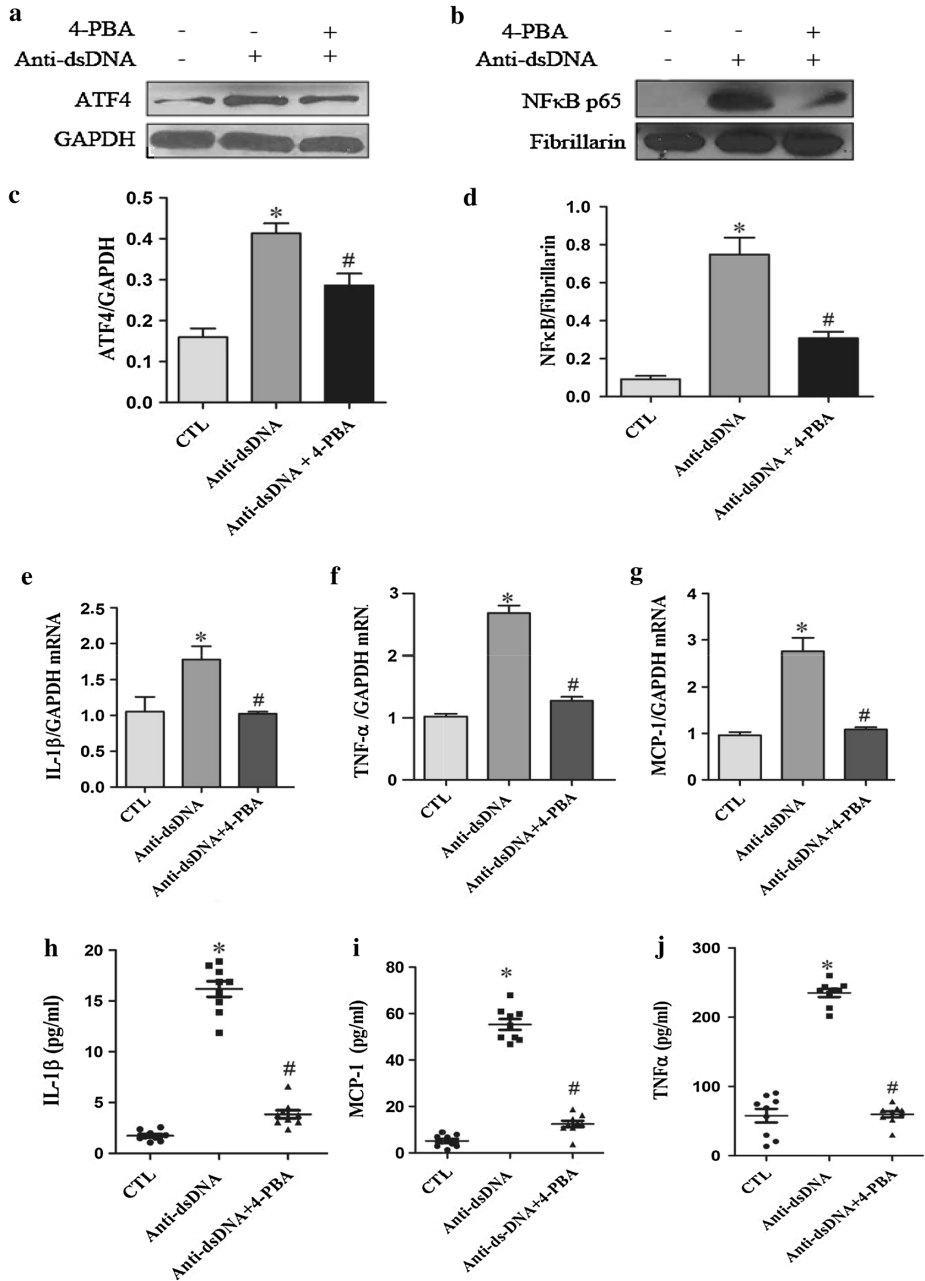
Chemical chaperone inhibited ER stress induced by anti-dsDNA antibodies in HMCs. **a**, **b** HMCs were stimulated with anti-dsDNA antibodies. The expression of ATF4 and nuclear translocation of NF-κB p65 were measure by western blot. **c**, **d** 4-PBA significantly inhibited the expression of ATF4 and the nuclear translocation of NF-κB p65 induced by anti-dsDNA antibodies. **e**–**g** 4-PBA significantly inhibited the expression of IL-1β, MCP-1 and TNF-α mRNA as measured by qPCR. **h**–**j** 4-PBA significantly inhibited the secretion of IL-1β, MCP-1 and TNF-α in the supernatants as measured by ELISA. All experiments were repeated for three times. Data were expressed as mean ± SD, *P < 0.01, versus control media, #P < 0.01, versus anti-dsDNA antibodies group. *CTL* Control and *anti-dsDNA Abs* anti-dsDNA antibodies.

HMCs were exposed to anti-dsDNA antibodies for 24 h. An ER stress inhibitor, the chemical chaperone 4-PBA, was included in some of the experiments. The expression of ATF4 in HMCs stimulated with anti-dsDNA antibodies was significantly inhibited by 4-PBA (Figure 5a). NF-κB p65 nuclear translocation in HMCs stimulated with 4-PBA was also significantly inhibited by 4-PBA (Figure 5b). Furthermore, 4-PBA treatment resulted in significant reduction in the expression of IL-1β, TNF-α and MCP-1 mRNA (Figure 5e–g) and their secretions in supernatants (Figure 5h–j).

### Anti-dsDNA antibodies induced the activation of NF-κB in HMCs via PERK-eIF2α-ATF4 pathway

**Figure 6:**
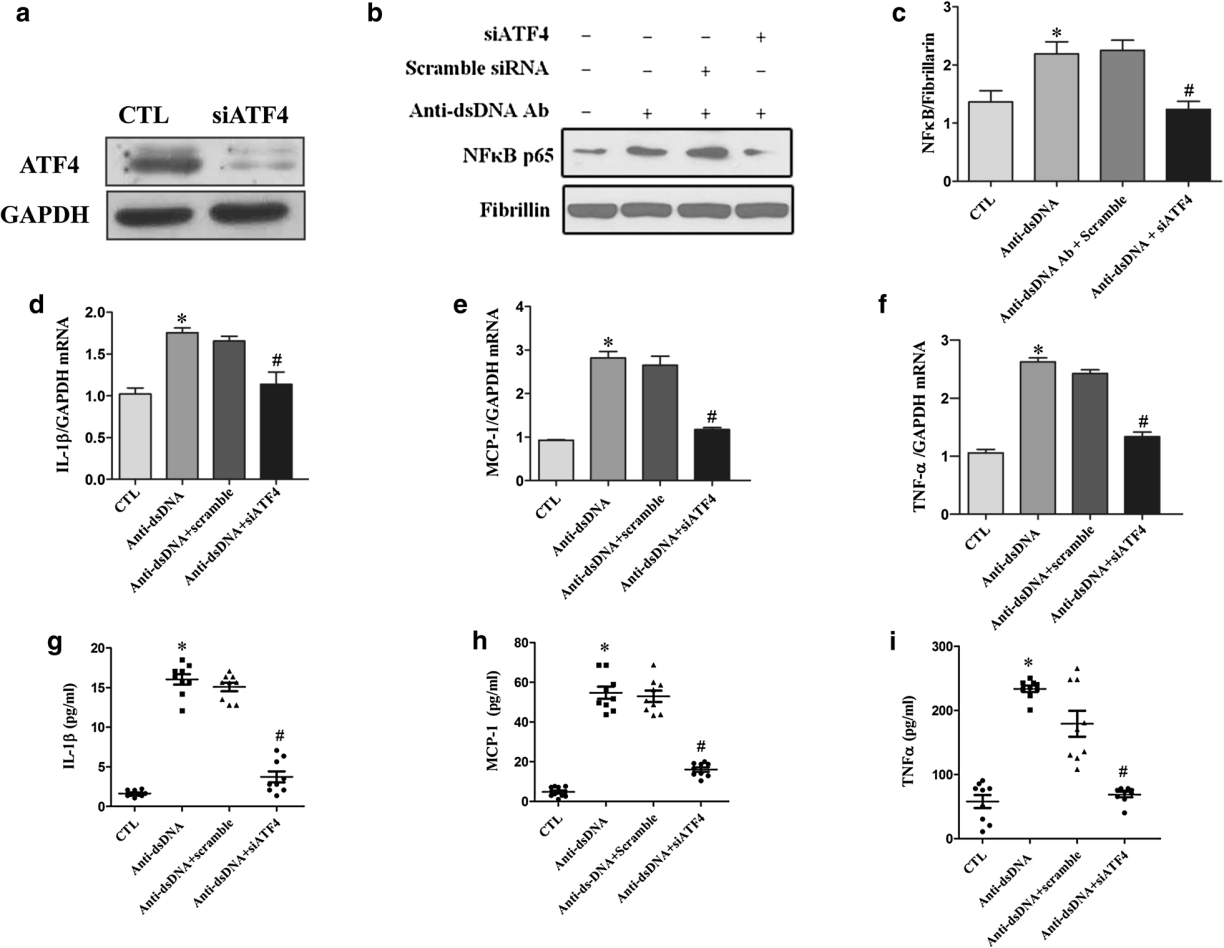
Anti-dsDNA antibodies induced NF-κB activation via PERK-eIF2α-ATF4 ER stress pathway. **a** Depletion of ATF4 by ATF4 siRNA was confirmed by western blot. **b** HMCs were stimulated with anti-dsDNA antibodies. NF-κB p65 nuclear translocation was measured by western blot analysis of nuclear protein extract. **c** ATF4 siRNA significantly inhibited NF-κB p65 nuclear translocation induced by anti-dsDNA antibodies. **d**–**f** ATF4 siRNA significantly inhibited the expression of IL-1β, MCP-1 and TNF-α mRNA as measured by qPCR. **g**–**i** ATF4 siRNA also significantly inhibited the secretion of IL-1β, MCP-1 and TNF-α in supernatants as measured by ELISA. All experiments were repeated for three times. Data were expressed as mean ± SD, *P < 0.01, versus control group, #P < 0.01, versus scramble siRNA group. *CTL* Control and *anti-dsDNA Abs* anti-dsDNA antibodies.

HMCs were transfected with siRNA specific for ATF4 or control siRNA. The depletion of ATF4 was confirmed by western blot analysis (Figure 6a). Specific depletion of ATF4 suppressed the nuclear translocation of NF-κB p65 induced by anti-dsDNA antibodies (Figure 6b). In addition, depletion of ATF4 significantly reduced anti-dsDNA antibody-induced expression of IL-1β, MCP-1 and TNF-α (Figure 6d–f), as well as the secretion of IL-1β, MCP-1 and TNF-α in the supernatant of HMCs (Figure 6g–i).

## Discussion

This study demonstrated that anti-dsDNA antibodies can bind to HMCs and result in NF-κB activation and increased the expression of pro-inflammatory cytokines through the induction of ER stress. The anti-dsDNA antibody-induced inflammation in HMCs was mediated through the PERK-eIF2α-ATF4 ER stress pathway.

Although anti-dsDNA antibodies are believed to be involved in the pathogenesis of LN, how they deposit and elicit inflammation in the glomeruli is not fully elucidated. Early study showed that a monoclonal anti-dsDNA antibody PME77 bound to a number of human cell types: erythrocytes, neuroblastoma, a T-lymphoblastoid cell line (HSB2), a B-lymphoblastoid cell line (Ramos) and normal T and B lymphocytes [22]. Subsequent study revealed that anti-dsDNA antibody bind to cell surface protein via nuceleosomes or a DNA-histone complexs in human fibroblast CVI cells [23]. In human mesangial cells (HMCs), affinity-purified polyclonal anti-DNA antibodies from patients with SLE can bind to cultured HMCs [21], which was mediated though annexin II [5]. It is interesting to note that the binding of anti-DNA antibodies to HMC was increased after the removal of Ig-associated DNA by DNase treatment, but it was unaffected by DNase treatment of HMC membrane [21]. These suggest a direct binding of anti-dsDNA antibodies to HMC rather than mediation through DNA or DNA-histone complex. In the present study, binding of anti-dsDNA antibodies to HMCs was confirmed by flow cytometry and by cellular ELISA. In addition, blocking of Fc receptor did not affect the binding of anti-dsDNA antibodies to HMCs, indicating that anti-dsDNA antibodies bind to HMCs via Fab fragments.

ER stress has been reported to be associated with chronic inflammatory or autoimmune diseases including diabetes and obesity, neurodegenerative and neuromuscular inflammatory diseases, and inflammatory bowel diseases [24–26]. ER stress is also observed in renal inflammation [27, 28]. In a rat anti-Thy1 mesangioproliferative nephritis, there was an increase in the expression of the ER stress-inducible chaperones GRP78 and oxygen-related protein 150 in isolated glomeruli, especially in the glomerular epithelial cells and mesangial cells, after the induction of the disease [29]. In human primary glomerulonephritis (GN), there was pronounced increased expression of GRP78 in proliferative GN compared to non-GN, suggesting that ER stress pathway might be involved in the progression of GN [30]. The role of ER stress in lupus nephritis (LN) is still not clear. It has been shown that homocysteine-induced ER protein (Herp), an ER stress-inducible protein, was able to bind to anti-dsDNA antibodies [31], and was a potential triggering antigen for anti-DNA response [32], implying that anti-dsDNA antibodies have a functional connection with ER stress in the pathogenesis of LN. In this study, we found that stimulation of HMCs with anti-dsDNA antibodies resulted in significantly increased expression of GRP78 and increased expression of inflammatory cytokines IL-1β, MCP-1 and TNF-α. These results suggest that anti-dsDNA antibodies can elicit proinflammatory response in HMC via inducing ER stress.

The ER stress response consists of three transmembrane signal transducers including protein kinase IRE1, PERK and the transcription factor ATF6 [10]. Our results showed that the ER stress inducer thapsigargin can activate all three pathways. In contrast, anti-dsDNA antibodies only significantly activate PERK and its downstream molecules, eIF2α and ATF4. There were no significant changes in the expression of p-IRE1α or ATF6, indicating anti-dsDNA antibodies elicit ER stress in HMCs through PERK pathway only. The reason for such a discrepancy is not clear. It is postulated that anti-dsDNA antibodies might bind to the membrane molecule that activates PERK ER stress pathway specifically.

NF-κB, a major transcription factor in regulating inflammatory processes, is involved in the pathogenesis of LN. Inhibition of NF-κB resulted in ameliorated inflammation in the mouse LN model [33]. It has been shown that ER stress induces the activation of NF-κB [34–36]. ER stress inducers including thapsigargin and tunicamycin increase the activity of NF-κB as well as NF-κB-dependent gene expression [37]. PERK and eIF2α were reported to be involved in the activation of NF-κB and the phosphorylation of PERK and eIF2α were in accordance with the activation of IKK/NF-κB [36]. This implies that PERK pathway might play an important role in the activation of NF-κB. In the present study, anti-dsDNA antibodies induced the activation of ER stress accompanied with the activation of NF-κB and up-regulation of IL-1β, TNF-α and MCP-1. Inhibition of ER stress in HMCs with chemical chaperon 4-PBA reduced the activation of NF-κB and the expression of IL-1β, TNF-α and MCP-1. In addition, specific depletion of ATF4 significantly reduced the activation of NF-κB and the expression of proinflammatory cytokines. These indicate that anti-dsDNA antibodies activate NF-κB in HMC via inducing ER stress.

The activation of CHOP in ER stress is recognized to be involved in cell apoptosis [38]. However, CHOP can also be activated by ATF4 and increase the production of inflammatory cytokines [39]. These raise the the possibility that the upregulation of proinflammatory cytokines induced by anti-dsDNA antibodies in HMCs is the result of CHOP activation rather than through NF-κB activation. However, inhibition of ER stress by chemical chaperon or the specific ATF4 silencing did reduce NF-κB activation, suggesting a cross talk between ER stress signaling pathway and NF-κB activation. Indeed, ATF4 is involved in the activation of NF-κB in macrophage induced by saturated fatty acids [40]. The activation of NF-κB was significantly decreased in ATF4 haploinsufficiency macrophage [40]. On the other hand, our results showed that anti-dsDNA antibodies did not induce the expression of CHOP. This study provides new evidences that anti-dsDNA antibodies activate NF-κB and cause inflammation in HMCs via PERK-eIF2α-ATF4 ER stress pathway.

## Conclusions

This study demonstrated that anti-dsDNA antibodies induce NF-κB activation and enhanced expression of pro-inflammatory cytokines in HMCs via PERK-eIF2α-ATF4 ER stress pathway. ER stress response may be involved in the development or progression of LN initiated by anti-dsDNA antibodies.
